# Comparison of planning techniques when air/fluid is present using the strut‐adjusted volume implant (SAVI) for HDR‐based accelerated partial breast irradiation

**DOI:** 10.1120/jacmp.v14i6.4442

**Published:** 2013-11-04

**Authors:** Joseph F. Harmon, Brandon K. Rice

**Affiliations:** ^1^ Department of Radiation Oncology Bon Secours Cancer Institute Henrico VA USA

**Keywords:** SAVI, HDR, APBI, breast cancer, brachytherapy

## Abstract

The presence of air/fluid surrounding implantable devices used for partial breast irradiation may significantly impact dose coverage to at‐risk tissue. Of the 67 total patients retrospectively evaluated for this study, 32 (48%) had greater than 1 cc volume of air/fluid extending outside of the strut‐adjusted volume implant (SAVI) device surface and were selected for comparison of planning approaches. The planning approaches utilized two different definitions of PTV_EVAL. One definition of a PTV_EVAL $\left({\text{PTV}\_\text{EVAL}}_{\text{SAVI}}\right)$ was based on expanding 1 cm beyond the SAVI device only while accounting for the air/fluid using the NSABP Protocol B‐39/RTOG Protocol 0413. The second PTV_EVAL definition $\left({\text{PTV}\_\text{EVAL}}_{\text{CAV}}\right)$ was based on expanding 1 cm beyond the cavity (SAVI device plus air/fluid volume). The results indicate use of the B‐39 formalism to account for air/fluid displacing the PTV_EVAL may overestimate the dose coverage to the at‐risk tissue, especially for large contiguous volumes of air/fluid. Using the SAVI device to optimize dose covering the ${\text{PTV}\_\text{EVAL}}_{\text{CAV}}$ volume surrounding the cavity improves dosimetric coverage to at‐risk tissue by 11.3% and 8.7% for V100 and V90, respectively, while the average V150 and V200 indices for ${\text{PTV}\_\text{EVAL}}_{\text{CAV}}$ increased by 9.1 cc and 5.0 cc, respectively, and the average maximum rib and skin doses increased by 11. 1% and 6.1%, respectively. The maximum skin dose, rib dose, V150, and V200 all met the planning objectives despite any increase in these parameters.

PACS number: 87.55.kh

## I. INTRODUCTION

Whole or partial breast irradiation (FBI) methods are clinically available for breast conservation therapy following lumpectomy, with accelerated partial breast irradiation (APBI) an option for many patients. Accelerated partial breast irradiation is typically delivered using high‐dose‐rate (HDR) brachytherapy or using 3D conformal radiotherapy (3D CRT), and delivers a higher dose per fraction over a shorter period of time to the tumor bed plus a 1–2 cm margin.^(1)^ HDR‐based approaches include multicatheter interstitial, balloon based systems, hybrid brachytherapy systems, and focused external irradiation.^(2)^

Multicatheter interstitial therapy dates back several decades and has the most mature clinical data.^1^, ^2^, ^3^, ^4^, ^5^, ^6^, ^7^, ^8^, ^9^, ^10^, ^11^, ^12^, ^13^, ^14^, ^15^ The typical dose of 34 Gy in 10 fractions twice daily (BID) for HDR was based on equivalence of the biological effective dose (BED) of a low‐dose rate interstitial delivery of 45 Gy in 4.5 days used in early AFBI trials.^(1)^

Examples of balloon‐based systems include Ir‐192 based MammoSite^(^ ^1^ ^,^ ^2^ ^,^ ^15^, ^16^, ^17^, ^18^, ^19^, ^20^ ^)^ (Cytyc Surgical, Falo Alto, CA), Contura^12^, ^21^ (SenoRX, Tempe, AZ), and low photon energy‐based electronic brachytherapy such as Axxent^(^ ^1^ ^,^ ^2^ ^)^ (Sound‐Eklin, Carlsbad, CA). Examples of hybrid brachytherapy systems include SAVI^20^, ^21^, ^22^, ^23^, ^24^ (Cianna Medical, Aliso Viejo, CA) and ClearFath^(1)^ (North American Scientific Inc., Chatsworth, CA).

NSABF Protocol B‐39/RTOG Protocol 0413^(25)^ compares whole breast irradiation (WBI) to FBI, including multicatheter interstitial and single‐entry intracavitary devices such as MammoSite, MammoSite ML, Contura, and SAVI. For the single entry intracavitary devices the protocol specifies a 1 cm expansion beyond the balloon/device surface to generate the PTV_EVAL excluding the pectoralis muscles, chest wall, and the first 5 mm beneath the skin. The protocol allows for air and/or fluid displacing some of the at‐risk tissue away from the FTV‐EVAL volume without requiring any modification of the FTV_EVAL. Typically when the volume of trapped air/fluid is less than 10% of the FTV_EVAL, acceptable dose coverage can be achieved. A recent study^(26)^ focused on the dosimetric effects of air pocket sizes outside of MammoSite balloons, providing support for the 10% rule commonly used per the NSABF Protocol.

An alternate planning approach to account for tissue displaced by air and/or fluid defines PTV_EVAL as a 1 cm thick shell encompassing the cavity rather than the balloon or device where the cavity is defined as the volume including the balloon or device plus the air/seroma. This approach should offer improved dose coverage for at‐risk tissue, but may adversely impact the dose to normal tissue such as ribs and skin. Also, providing adequate dose conformity and dose homogeneity for the irregular shape of the FTV_EVAL encompassing the cavity may be quite difficult for most single entry intracavitary devices.

A recent study^(21)^ evaluated the capability of both the Contura and SAVI devices to treat asymmetric volumes of breast tissue where the margins varied from as small as 0.3 cm to as large as 1.5 cm. The researchers concluded the SAVI device demonstrated the greatest dosimetric flexibility, especially when targeting margins greater than 1.1 cm. Although the asymmetric margins used in this study were not irregular in shape as is commonly seen in the clinic, the research did show promise for using an asymmetrical FTV_EVAL expansion approach.

The focus of this work is to retrospectively compare both FTV_EVAL expansion approaches for the SAVI applicator for a large number of patient plans. Dual approach planning for each CT dataset, along with the variety afforded by a large number of patients, provides an excellent opportunity to characterize and contrast dosimetric parameters for the two approaches in a clinically relevant manner.

## II. MATERIALS AND METHODS

### A. Patient selection

The SAVI applicator is available in four sizes to accommodate various cavity volumes: the 6–1, 6–1 mini, 8–1, and 10–1, and their physical characteristics have been described in the literature.(23) Patients implanted with any size of SAVI device were considered for this work, including patients with a device‐to‐skin distance less than 5 mm. A total of 67 consecutive partial breast patients treated with the SAVI applicator between February 2011 and September 2012 were analyzed to determine the subset of patients with air and/or fluid volume greater than 1cc outside of the device surface. Of the 67 total patients treated, 32 (48%) had greater than 1cc volume of air/fluid extending outside of the SAVI device surface and were included in this study. Of the 32 patients included in the study, 26 had air/fluid volume ranging from 1 to 5 cc, with the remainder (six) having air/fluid exceeding 5 cc. Two patients were implanted with the 6–1 mini, eight patients with the 6–1, 13 patients with the 8–1, and nine patients with the 10–1. Retrospective treatment planning was performed on this group of 32 patients to compare the results of different planning approaches.

### B. Treatment planning technique

Oncentra Version 4.1 (Elekta, Stockholm, Sweden) was used for treatment planning. The initial planning CT datasets from the selected group of SAVI patients with 1 cc or greater air/fluid volume were used to explore planning results obtained from two unique target volumes. The first target volume, ${\text{PTV}\_\text{EVAL}}_{\text{savi}}$, was created by uniformly expanding the contoured SAVI device structure 1 cm in all directions and then subtracting the SAVI structure from the expanded volume. The ${\text{PTV}\_\text{EVAL}}_{\text{SAVI}}$ was limited to the posterior breast tissue extent as described in the B‐39 protocol,^(25)^ but we chose to bound the ${\text{PTV}\_\text{EVAL}}_{\text{SAVI}}$ by 2 mm from the skin surface to be consistent with what is performed in practice at our clinic. The volume of air or fluid outside of the SAVI device was contoured but not used to physically modify the ${\text{PTV}\_\text{EVAL}}_{\text{SAVI}}$ volume. The ribs, pectoralis muscle, skin, and lung were contoured for all cases, with the skin defined as the first 2 mm depth of the body. To create the second target structure, air/fluid existing outside of the SAVI device bounds was combined with the SAVI device volume to create the true cavity structure. The target volume ${\text{PTV}\_\text{EVAL}}_{\text{CAV}}$ was created by expanding the cavity 1 cm in all directions, subtracting the cavity volume, and limiting the volume to the posterior breast tissue extent and skin as was done with the ${\text{PTV}\_\text{EVAL}}_{\text{SAVI}}$ structure.

The two target volumes ${\text{PTV}\_\text{EVAL}}_{\text{SAVI}}$ and ${\text{PTV}\_\text{EVAL}}_{\text{CAV}}$ were used to create a total of three unique dosimetric planning scenarios for each patient. The following dosimetric indices were calculated for each case: V100%, V90%, V150(cc), V200(cc), dose homogeneity index (DHI), dose conformity index (DCI), and maximum skin and rib dose (defined as the maximum dose to 0.01 cc volume).

The DHI is defined as:^(25)^

(1) DHI=1−V150(cc)/V100(cc)

(2) $$\text{DCI}={\left({\text{TV}}_{\text{RI}}\right)}^{2}/(\text{TV}\times V_{\text{RI}})$$

where, ${\text{TV}}_{\text{RI}}=\text{target volume covered by the reference isodose}\;(\text{cc}),\;\text{TV}=\text{target volume}\;(\text{cc}),\;V_{\text{RI}}=\text{volume of reference isodose}\;(\text{cc})$.

**Figure 1:**
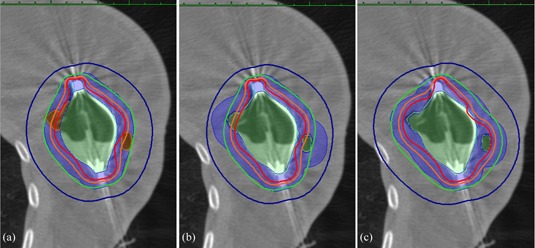
Illustrations of (a) ${\text{PTV}\_\text{EVAL}}_{\text{SAVI}}$, represented as the blue shaded region, for the Scenario #1 planning approach; (b) ${\text{PTV}\_\text{EVAL}}_{\text{CAV}}$, represented as the blue shaded region, for the Scenario #2 planning approach; (c) ${\text{FTV}\_\text{EVAL}}_{\text{CAV}}$ represented as the blue shaded region, for the Scenario #3 planning approach. The green line represents 100% isodose.

In the first planning scenario, dosimetric coverage was optimized to the ${\text{PTV}\_\text{EVAL}}_{\text{SAVI}}$ target volume, as illustrated in Fig. 1(a). This represents the traditional approach to planning, where the volume of air/fluid trapped outside of the SAVI device is ignored during the 1 cm expansion of the cavity (SAVI) to create the ${\text{PTV}\_\text{EVAL}}_{\text{SAVI}}$, but is used to modify the dosimetric parameters V90% and V100%, per the B‐39 formalism:^(25)^

(3) $$\begin{array}{l} \text{Corrected}\;\%\text{PTV}\_\text{EVAL}\;\text{Coverage}=\\ \%PTV\_EVA\text{LC}overage-\frac{Vo\text{lT}rappe\text{da}ir/fluid}{Vo\text{lP}TV_{EVAL}}\times 100 \end{array}$$

The use of the above formula relies on the assumption that the volume of trapped air/fluid present equals the amount of FTV_EVAL displaced.

The second planning scenario, illustrated in Fig. 1(b), was created by superimposing the ${\text{PTV}\_\text{EVAL}}_{\text{CAV}}$ target volume onto the dose distribution obtained from the traditional planning Scenario #1 approach. This situation characterizes the dose actually delivered to ${\text{PTV}\_\text{EVAL}}_{\text{CAV}}$ (based on the 1 cm expansion of the cavity including the SAVI+air/fluid volume) when optimizing the dose to the ${\text{PTV}\_\text{EVAL}}_{\text{savi}}$.

Finally, a third planning scenario was created by optimizing the dosimetric coverage of the ${\text{PTV}\_\text{EVAL}}_{\text{CAV}}$ target volume, as illustrated in Fig. 1(c). This method optimizes the dose to the ${\text{PTV}\_\text{EVAL}}_{\text{CAV}}$ target volume encompassing the SAVI and air/fluid volumes comprising the cavity. In this scenario, no formula is used to amend the V90% and V100% based on the volume of trapped air/fluid as is needed in the traditional planning Scenario #1 approach.

The same approach to optimizing the dose distribution was carried out for all cases. First, only the central catheter is loaded with 5 mm step‐size dwell points throughout the entire length of the device. Dose is prescribed as 34.0 Gy in 10 fractions to a point on the outer surface of the PTV_EVAL. Graphical optimization is used to create an ellipsoidal shape of dose that best covers the target. Next, the outer catheters are then activated with zero time assigned to them. Finally, graphical optimization is used engage the outer catheters and to fine tune the plan until planning objectives are met. Our treatment planning objectives are outlined in Table 1.

**Table 1:** Treatment planning objectives

*Dosimetric Index (units)*	*Planning Goal*
V100 (%)	Goal >95%, but >85% acceptable
V90 (%)	Goal >99%, but >90% acceptable
V150 (cc)	<50cc
V200 (cc)	<20cc
Max. (0.01 cc) Skin Dose (%)	Goal <125% of prescribed dose, but <145% acceptable
Max. (0.01 cc) Rib Dose (%)	Goal <125% of prescribed dose, but <145% acceptable

### C. Statistical analysis

Paired, two‐tail, *t*‐tests were performed for all dosimetric index differences to determine if they were significant. A p‐value less than or equal to 0.05 was considered statistically significant for this study. A Pearson's correlation analysis was performed for percent changes in dosimetric indices as a function of absolute air/fluid volume.

## III. RESULTS & DISCUSSION

### A. ${\text{PTV}\_\text{EVAL}}_{\text{SAVI}}$ (planning Scenario #1) vs. ${\text{PTV}\_\text{EVAL}}_{\text{CAV}}$ (planning Scenario #2)

**Figure 2:**
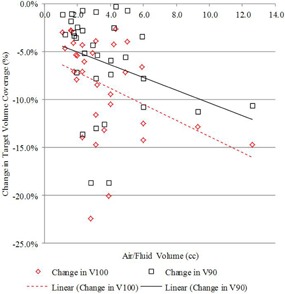
Change in V100% and V90%, comparing ${\text{PTV}\_\text{EVAL}}_{\text{SAVI}}$ (planning Scenario #1) vs. ${\text{PTV}\_\text{EVAL}}_{\text{CAV}}$ (planning Scenario #2), as a function of air/fluid volume.

**Figure 3:**
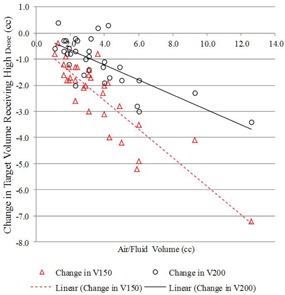
Change in V150(cc) and V200(cc), comparing ${\text{PTV}\_\text{EVAL}}_{\text{SAVI}}$ (planning Scenario #1) vs. ${\text{PTV}\_\text{EVAL}}_{\text{CAV}}$ (planning Scenario #2), as a function of air/fluid volume.

The first and second treatment planning scenarios share the same dose distribution but different planning target volumes. Such a comparison is useful to illustrate the lower dose coverage experienced by ${\text{PTV}\_\text{EVAL}}_{\text{CAV}}$ when utilizing the traditional planning Scenario #1 approach for ${\text{PTV}\_\text{EVAL}}_{\text{SAVI}}$. Differences in FTV_EVAL coverage between the first two planning scenarios also help determine how well the B‐39 formalism accounted for the displacement of the PTV_EVAL by trapped air/fluid, with results summarized in Table 2. A decrease in target coverage for all patients was observed between the ${\text{PTV}\_\text{EVAL}}_{\text{SAVI}}$ and ${\text{PTV}\_\text{EVAL}}_{\text{CAV}}$ volumes when the traditional planning approach was used to optimize the target coverage on ${\text{PTV}\_\text{EVAL}}_{\text{SAVI}}$ with the B‐39 formalism. The mean V100% and V90% decreased by 8.5% and 6. 1%, respectively. The mean V150 and V200 also showed a slight decrease; this result was expected because the air/fluid encompassed by the ${\text{PTV}\_\text{EVAL}}_{\text{SAVI}}$ was not considered part of the ${\text{PTV}\_\text{EVAL}}_{\text{CAV}}$. All results were shown to be statistically significant. Figure 2 illustrates the change in V100% and V90% between the plans as a function of air/fluid volume. Results showed that an increasing volume of air/fluid was weakly negatively correlated (−0.39and−0.31) with decreasing difference in V100% and V90% between the two plans. An increasing volume of air/fluid was shown to be strongly negatively correlated (−0.87and−0.73) with decreasing V150(cc) and V200(cc), as can be seen in Fig. 3.

**Table 2:** Dosimetric comparison of ${\text{PTV}\_\text{EVAL}}_{\text{SAVI}}$ and ${\text{PTV}\_\text{EVAL}}_{\text{CAV}}$ for planning Scenarios #1 and #2

*Mean Dosimetric Index (units)*	${\text{PTV}\_\text{EVAL}}_{\text{SAV}}$, *(Planning Scenario #)*	${\text{PTV}\_\text{EVAL}}_{\text{CAV}}$ *(Planning Scenario #2)*	*Average Difference*	*P‐value*
V100 (%)	90.9	82.4	−8.5	1.7E‐10
V90 (%)	94.6	88.5	−6.1	1.4E‐7
V150 (cc)	31.7	29.3	−2.4	3.7E‐10
V200 (cc)	12.4	11.3	−1.1	1.4E‐7
DHI	0.56	0.57	+0.01	2.2E‐4
DCI	0.89	0.76	−0.12	1.7E‐11

**Figure 4:**
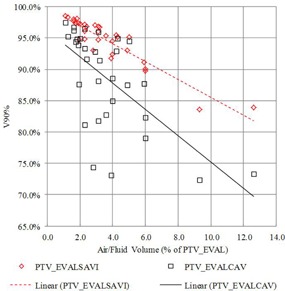
Relationship between the calculated V90% and air/fluid volume (as a percentage of ${\text{PTV}\_\text{EVAL}}_{\text{SAVI}}$) for ${\text{FTV}\_\text{EVAL}}_{\text{SAVI}}$ (planning Scenario #1) and ${\text{PTV}\_\text{EVAL}}_{\text{CAV}}$ (planning Scenario #2).

Considering the ${\text{PTV}\_\text{EVAL}}_{\text{SAVI}}$ target volume alone, treatment plans for 3/32 (9.4%) patients would not have been acceptable for treatment (per the B‐39 protocol) because the air/fluid volume corrected V90 was less than 90%. If the ${\text{PTV}\_\text{EVAL}}_{\text{CAV}}$ target volume was considered, 15/32 (47%) patients would not have met the criteria for appropriateness for treatment. Figure 4 shows the relationship between the calculated V90% and air/fluid volume (as a percentage of ${\text{PTV}\_\text{EVAL}}_{\text{SAVI}}$) for both target volumes. The data suggest the treatment planning objective V90>90% cannot be achieved when the air/fluid volume is greater than roughly 7% of ${\text{PTV}\_\text{EVAL}}_{\text{SAVI}}$ volume for the ${\text{PTV}\_\text{EVAL}}_{\text{SAVI}}$. The data for ${\text{PTV}\_\text{EVAL}}_{\text{CAV}}$ are more widely dispersed, presumably due to the large variability of size and location of the trapped air/fluid and its impact on dosimetric coverage. Even so, it can be seen that a large disparity exists between the real amount of FTV_EVAL displaced by air/fluid and what is calculated using the B‐39 formalism. When evaluating ${\text{PTV}\_\text{EVAL}}_{\text{cav}}$, no patient had V90>90% whose air/fluid volume was above 5%.

### B. ${\text{PTV}\_\text{EVAL}}_{\text{CAV}}$ (planning Scenario #2) vs. ${\text{PTV}\_\text{EVAL}}_{\text{CAV}}$ (planning approach Scenario #3)

**Figure 5:**
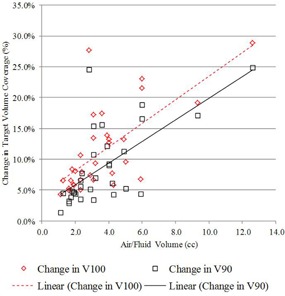
Change in ${\text{PTV}\_\text{EVAL}}_{\text{CAV}}$ V100% and V90%, comparing planning Scenarios #2 and #3 with increasing air/fluid volume.

**Figure 6:**
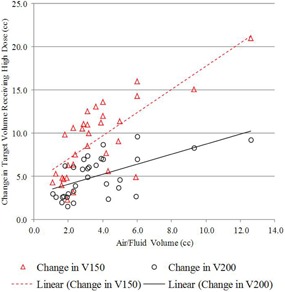
Change in ${\text{PTV}\_\text{EVAL}}_{\text{CAV}}$ V150(cc) and V200(cc), comparing planning Scenarios #2 and #3 with increasing air/fluid volume.

**Figure 7:**
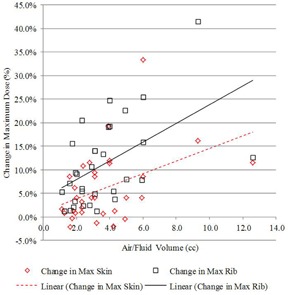
Change in maximum skin and rib dose, comparing planning Scenarios #2 and #3 with increasing air/fluid volume.

The second and third treatment planning scenarios have different dose distributions but share the same target volume, which permits a side‐by‐side comparison of identical at‐risk tissue planned using two different approaches. The second and third treatment scenarios were compared to determine whether dosimetric coverage could be improved by optimizing the treatment plan to the ${\text{PTV}\_\text{EVAL}}_{\text{CAV}}$ volume and evaluate the resulting impact on skin and rib dose, and DHI and DCI. As shown in Table 3, the average ${\text{PTV}\_\text{EVAL}}_{\text{CAV}}$ coverage increased by 11.3% and 8.7% for V100 and V90, respectively. Target coverage increased in all patients when the plan was optimized to cover the ${\text{PTV}\_\text{EVAL}}_{\text{CAV}}$ target volume. The increase in target tissue coverage is shown graphically in Fig. 5 for V100 and V90 where both dose‐volume index changes are strongly positively correlated (0.68 and 0.68, respectively) with increasing air/fluid volume. Along with the increased dosimetric coverage came an increase in V150 and V200 for all patients. The average V150 and V200 indices for ${\text{PTV}\_\text{EVAL}}_{\text{CAV}}$ increased by 9.1 cc and 5.0 cc, respectively. The increase in high dose in the target tissue volume is shown graphically in Fig. 6 for V150 and V200 where both dose‐volume index changes are strongly positively correlated (0.74 and 0.59, respectively) with increasing air/fluid volume. The increased V150 is consistent with decreased DHI for all patients. The rib dose was shown to increase for all patients and the skin dose increased for 27/32 (84%) patients. The average maximum rib and skin doses increased by 11.1% and 6.1%, respectively. Figure 7 shows the relationship between the change in maximum skin and rib dose as a function of air/fluid volume. All results were shown to be statistically significant. The maximum skin dose, rib dose, V150, and V200 all met the planning objectives, despite any increase in these parameters.

**Table 3:** Comparison of ${\text{PTV}\_\text{EVAL}}_{\text{CAV}}$ dosimetric indices for planning Scenarios #2 and #3

*Mean Dosimetric Index (units)*	${\text{PTV}\_\text{EVAL}}_{\text{CAV}}$ *(Planning Scenario #2)*	${\text{PTV}\_\text{EVAL}}_{\text{CAV}}$ *(Planning Scenario #3)*	*Average Difference*	*P‐value*
V100 (%)	82.4	93.7	+11.3	1.13E‐10
V90 (%)	88.5	97.2	+8.7	8.36E‐09
V150 (cc)	29.3	38.4	+9.1	4.12E‐13
V200 (cc)	11.3	16.3	+5.0	4.12E‐13
Max Skin (%)	96.5	102.6	+6.1	5.88E‐05
Max Ribs (%)	91.2	102.3	+11.1	9.55E‐08
DHI	0.57	0.51	−0.06	1.96E‐10
DCI	0.76	0.78	+0.02	1.24E‐02

Most of the patients whose ${\text{PTV}\_\text{EVAL}}_{\text{CAV}}$ V90% coverage was less than 90% with the traditional planning approach (planning Scenario #2) were able to be salvaged using the planning Scenario #3 method. Twelve of the 15 patients (80%) replanned by optimizing dose to ${\text{PTV}\_\text{EVAL}}_{\text{CAV}}$ saw the V90% increase to or above 90% while still meeting or exceeding the other planning objectives. There are several variables that may have an influence on the newly planned dosimetric coverage of ${\text{PTV}\_\text{EVAL}}_{\text{cav}}$. Aside from the volume of trapped air/fluid, the device‐to‐skin/rib distance, location and shape of the trapped air/fluid relative to the SAVI device, and continuity of trapped air/fluid are all factors that can affect the ability to plan adequate coverage and are beyond the scope of this work.

### C. ${\text{PTV}\_\text{EVAL}}_{\text{SAVI}}$ (planning Scenario #1) vs. ${\text{PTV}\_\text{EVAL}}_{\text{CAV}}$ (planning Scenario #3)

Although the first and third treatment planning scenarios do not share dose distributions nor target volumes, we felt it was relevant to compare the two since the tabulated dose‐volumes indices are what would be reported clinically for either of the two planning strategies. As shown in Table 4, FTV_EVAL coverage increased by 2.8% and 2.6% for V100 and V90, respectively. The V150 and V200 indices for FTV_EVAL increased as well by 6.8 cc and 3.9 cc, respectively. The dose homogeneity index (DHI) decreased by 0.05 and the DCI decreased by 0.11. Recall, the first planning scenario uses the B‐39 correction formalism to account for the presence of air/fluid within ${\text{PTV}\_\text{EVAL}}_{\text{SAVI}}$, whereas the third planning scenario for ${\text{PTV}\_\text{EVAL}}_{\text{CAV}}$ does not use, or need, a correction to the V90 and V100 indices to account for air/fluid. If the B‐39 correction was not applied to the ${\text{PTV}\_\text{EVAL}}_{\text{SAVI}}$ volume using planning Scenario #1, the V90 and V100 values would certainly be larger than those of ${\text{PTV}\_\text{EVAL}}_{\text{CAV}}$ using planning Scenario #3. All results were determined to be statistically significant.

**Table 4:** Comparison of ${\text{PTV}\_\text{EVAL}}_{\text{SAVI}}$ and ${\text{PTV}\_\text{EVAL}}_{\text{CAV}}$ for planning Scenarios #1 and #3

*Mean Dosimetric Index (units)*	${\text{PTV}\_\text{EVAL}}_{\text{SAVI}}$ *(Planning Scenario #1)*	${\text{PTV}\_\text{EVAL}}_{\text{CAV}}$ *(Planning Scenario #3)*	*Average Difference*	*P‐value*
V100 (%)	90.9	93.7	+2.8	5.53E‐04
V90 (%)	94.6	97.2	+2.6	8.77E‐04
V150 (cc)	31.7	38.4	+6.8	1.77E‐11
V200 (cc)	12.4	16.3	+3.9	3.83E‐11
DHI	0.56	0.51	−0.05	8.61E‐10
DCI	0.89	0.78	−0.11	3.02E‐10

## IV. CONCLUSIONS

Use of the B‐39 formalism to account for air/fluid displacing the PTV_EVAL may overestimate the dose coverage to the at‐risk tissue, especially for large contiguous volumes of air/fluid. The entire cavity, including trapped air/fluid present outside of the device, may be used to create the PTV_EVAL when planning partial breast. The decision to treat all or part of the tissue extending beyond trapped air/fluid is made by the radiation oncologist familiar with the surgicopathologic data on a case‐by‐case basis. Using the SAVI device to optimize dose covering the ${\text{PTV}\_\text{EVAL}}_{\text{CAV}}$ volume surrounding the cavity improves dosimetric coverage to at‐risk tissue by 11.3% and 8.7% for V100 and V90, respectively, while the average V150 and V200 indices for ${\text{PTV}\_\text{EVAL}}_{\text{CAV}}$ increased by 9.1 cc and 5.0 cc, respectively, and the average maximum rib and skin doses increased by 11.1% and 6.1%, respectively. The maximum skin dose, rib dose, V150, and V200 all met the planning objectives, despite any increase in these parameters.

## Supporting information

Supplementary Material
