# Supplementary figures and images for: Comparison of planning techniques when air/fluid is present using the strut‐adjusted volume implant (SAVI) for HDR‐based accelerated partial breast irradiation
Source: J Appl Clin Med Phys. 2013 Nov 4;14(6):264–73. doi: 10.1120/jacmp.v14i6.4442 (PMC5714640; doi:10.1120/jacmp.v14i6.4442)

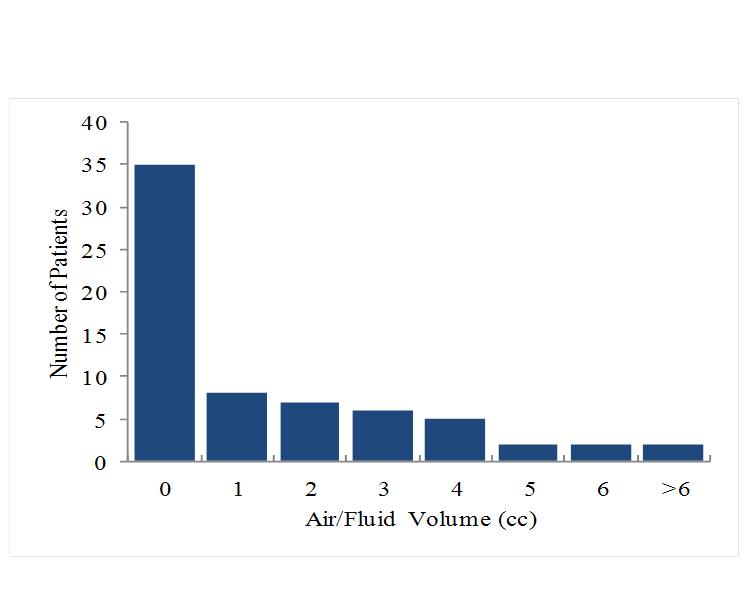

Supplement: Supplementary file 1 — Supplementary Material [file ACM2-14-264-s001.jpg]
